# Introducing virtual microscopy at the University of Ghana during the COVID‐19 pandemic—Identifying and overcoming obstacles in a low‐resource learning environment

**DOI:** 10.1002/ase.70276

**Published:** 2026-06-08

**Authors:** Nii Koney‐Kwaku Koney, Smart Nana Omenako, David Nana Adjei, Benjamin Arko‐Boham, Michael Hortsch

**Affiliations:** ^1^ Department of Anatomy University of Ghana Medical School, College of Health Sciences, University of Ghana Accra Ghana; ^2^ Department of Medical Laboratory Sciences, College of Health Sciences University of Ghana Accra Ghana; ^3^ Department of Cell and Developmental Biology University of Michigan Medical School Ann Arbor Michigan USA; ^4^ Department of Learning Health Sciences University of Michigan Medical School Ann Arbor Michigan USA

**Keywords:** COVID‐19, histology, microanatomy, student perception, sub‐Saharan Africa, virtual microscopy

## Abstract

The introduction of new educational technologies into resource‐challenged learning environments is often hindered by several factors, with the lack of infrastructure and computer hardware being only one critical aspect. The COVID‐19 pandemic required a sudden switch to online learning and accelerated the implementation of e‐learning approaches worldwide. This study follows the adoption of virtual microscopy (VM) during the COVID‐19 pandemic by medical and dental students at the University of Ghana (UG) for the learning of histology and the changes that were the result of this transformation. Students' awareness and the use of VM resources for their histology education increased significantly over the time of the pandemic. Students who were actively using VM reported a lack of internet access and insufficient promotion by histology teachers as major obstacles for their use of this educational technology. Although they attributed many advantages to VM as a histology learning tool, more than 90% of UG students advocated for the combined use of traditional and virtual microscopy during histology laboratory sessions. Students who did not use VM mostly blamed a lack of hardware devices, internet access, and technical knowledge for not making use of VM. In summary, although medical and dental students voiced strong support for the implementation of this e‐learning technology, a combination of factors including infrastructural limitations and a reluctance by teaching and administration staff might hinder the implementation of VM in a resource‐challenged learning environment like at the UG.

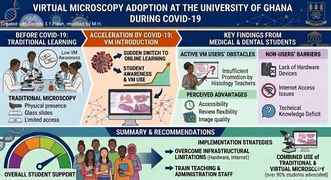

## INTRODUCTION

Histology or microanatomy is a fundamental preclinical component of medical and dental student education at African and other universities worldwide.^1^, ^2^, ^3^, ^4^ Students learn about the normal structure and functions of organs, tissues, cells, and subcellular organelles in metazoan animals and how they relate to disease processes. Therefore, histology is a foundational basic science and the starting point for understanding pathological changes at the cellular level.^1^, ^5^, ^6^ Usually, histology is taught as a combination of lecture‐style presentations and practical laboratory sessions, traditionally employing microscopy for visualizing structural features at the cellular level.^7^ For more than a century and a half, the laboratory learning component of histology education used light microscopy (LM) as its central technology.^8^, ^9^, ^10^ Since the turn of the millennium, this has dramatically changed due to the introduction of virtual microscopy (VM).^11^, ^12^, ^13^, ^14^, ^15^, ^16^, ^17^ For VM, high‐quality histology glass slides are scanned at high resolution and the resulting image files are available via local networks or the internet to histology learners using a computing device with appropriate viewer software.^1^, ^18^, ^19^ In contrast to static digital images, students can use a VM viewer software to select any area of a VM image at a magnification of their choosing. VM has gradually replaced traditional LM at most schools in North America, Europe, and Oceania.^3^, ^19^, ^20^, ^21^ This trend has also started at universities in many developing countries.^3^, ^19^

Numerous published studies have reported that VM delivers comparable if not superior learning outcomes for histology and pathology when compared to traditional LM instruction.^17^, ^22^, ^23^, ^24^, ^25^, ^26^, ^27^, ^28^ The educational use of VM offers several additional advantages, including superior and reproducible image quality, ease of sharing image files, enhanced accessibility, flexibility for the learning process, and opportunities for collaborative education.^15^, ^29^, ^30^, ^31^ Other studies found that VM also improves student engagement and the comprehension of histological and pathological concepts and that it is usually embraced by the learning community.^16^, ^32^, ^33^, ^34^, ^35^

Due to an unreliable internet infrastructure and lack of personal computing device ownership,^36^, ^37^ developing countries with a resource‐limited teaching environment trail affluent global regions in using VM for histology and pathology education.^3^, ^19^, ^38^, ^39^, ^40^, ^41^ Consequently, histology teaching at many African schools still relies on light microscopes and glass slides.^3^, ^19^, ^42^ Often, several students must share a single light microscope, and the upkeep of microscopes and replacement of broken glass slides are cumbersome and expensive, resulting in a suboptimal learning experience. Therefore, African histology teachers and learners are progressively embracing modern learning technologies including VM and other types of e‐learning strategies.^3^, ^19^, ^43^, ^44^ However, this progress has been very uneven on the African continent with universities in countries bordering the Mediterranean Sea and in South Africa using VM more frequently than those in other African countries.^45^, ^46^, ^47^, ^48^

As the in‐house development of virtual slide collections and the infrastructure to make them available to the local learner community requires considerable financial investments that are often beyond the means of a single educational institution,^18^, ^49^ VM use for histology laboratory instruction at African universities often relies on free websites that are offered by non‐African individuals and institutions.^50^, ^51^, ^52^

The COVID‐19 pandemic forced the rapid adoption of e‐learning strategies worldwide, including at African schools.^53^, ^54^, ^55^, ^56^, ^57^, ^58^, ^59^, ^60^, ^61^, ^62^ For histology teaching, this often involved a shift from traditional LM, which requires in‐person sessions, to online VM histology laboratory lessons. Reports from both affluent and economically challenged countries indicate that this COVID‐19‐induced switch was usually successful with some universities keeping VM as their main technology for histology laboratory instruction after pandemic restrictions were lifted.^56^, ^63^, ^64^, ^65^, ^66^, ^67^, ^68^

In this article, the authors report on the changes forced by the COVID‐19 pandemic to histology teaching of medical and dental students at the University of Ghana Medical School (UGMS)^69^ and University of Ghana Dental School (UGDS).^70^ Pre‐pandemic histology education at UGMS relied on traditional LM and glass slides. This article focuses on the identification of obstacles for the introduction of VM, mainly from the viewpoint of histology learners and their strategies of overcoming these challenges.

## MATERIALS AND METHODS

### Histology curriculum at the University of Ghana

The University of Ghana (UG) offers 6‐year undergraduate medical and dental programs that are modeled after the British school system^69^, ^70^ and a four‐and‐a‐half‐year Graduate Entry Medical Program (GEMP)^71^ that follows the American system. In the undergraduate program, the first year (level 100) provides general academic preparation, followed by integrated basic science and clinical courses from the second to the sixth year (levels 200–600). Medical and dental students are taught together for these first 3 years before separating into distinct clinical pathways (levels 400–600). The GEMP program enrolls students with prior undergraduate degrees into a shorter curriculum with three semesters in the first year where community health is introduced alongside basic sciences before the students join the undergraduate cohort for clinical training.^71^

Histology education for both medical and dental students is provided by the University of Ghana Medical School (UGMS). It is taught within a modular, system‐based structure. In the undergraduate program, it is integrated with gross anatomy, embryology, physiology, and pharmacology across modules such as musculoskeletal, gastrointestinal, genitourinary, and neuroscience (mostly at level 200), whereas the histology of blood and the lymphatic organs is taught in the clinical hematology module at the 300 level. In the GEMP program, histology is combined with gross anatomy and embryology in a stand‐alone human anatomy module in the first semester and neuroscience in the third semester. Didactic knowledge is delivered by 2‐h lectures, one per topic, that are complemented by 3‐h practical sessions using light microscopes and glass slides. Typically, four to five students share one light microscope. Students are provided with a printed text‐only manual for these laboratory sessions. This manual has not been revised for many years and makes no reference to outside supplemental learning resources. With the help of a histology textbook/atlas, students are supposed to follow the exercises described in the laboratory manual. Faculty members rotate between student groups to render assistance and answer questions. Due to the high student‐to‐microscope ratio, students are encouraged by the teaching faculty members to use outside virtual microscopy resources for augmenting their learning during practical sessions and for personal study. Some histology teachers also use VM during their lectures and supplement the LM laboratory component with the histology feature of the Anatomage table (Anatomage Inc., Santa Clara, CA, USA).^3^ However, the use of these VM resources is not encoded in the current curriculum and depends on the individual instructor. Students are assessed by multiple‐choice questions and practical “steeple chase” examinations that require the identification of structures under light microscopes or in printed micrographs. GEMP students, in addition, complete essay questions on histology concepts to reinforce the integration of basic science knowledge with clinical applications.

### Histology instruction at UGMS during the COVID‐19 pandemic

In response to the global COVID‐19 pandemic, the Ghanaian government declared a partial lockdown from April to July 2020 that mostly affected two major urban areas (Accra and Kumasi).^72^ Starting from March 16, 2020, the University of Ghana suspended all in‐person instructions.^54^, ^56^ Subsequently, histology lectures and laboratory sessions at UGMS were offered online via Zoom links (Zoom Communications, San Jose, CA, USA) or the Sakai learning platform.^73^ Students usually accessed the lectures and laboratory sessions using their own devices.

At the time when the survey was offered in 2023, the 200‐ and 300‐level students were actively engaging in histology learning. Higher class levels had completed histology education earlier, 1 year per higher class level. Students who were at the 500 level when the survey was offered during the early summer of 2023 had completed most of their histology education at the time of the pandemic outbreak in Ghana (March of 2020). Although the UGMC received a partial dispensation from the general lockdown protocol, this exemption was only used for clinical education and not for basic science instruction.

At the beginning of the pandemic in early 2020, histology instruction immediately transitioned to Zoom for all lectures, laboratory sessions, and student–faculty interactions. However, due to the rising cost of textbooks, microscope and slide shortages, and a more tech‐savvy student generation, the shift toward online resources had already started before the start of the pandemic. After an initial phase of online‐only histology instruction, starting on June 15, 2020, limited in‐person laboratory instruction with mandatory masking and smaller group sizes resumed for final‐year students. The UG computer facilities also reopened, although students were still advised to continue using their own computing devices.^59^ Starting with the 2021–2022 academic year, histology teaching at UGMS returned to the pre‐pandemic modus with glass slides and microscopes for histology laboratory instruction. However, after the return to the pre‐pandemic teaching curriculum, based on the experiences during the COVID‐19 pandemic and with a decreasing number of available functional microscopes and glass slide collections, UGMS histology learners, encouraged by UGMC faculty members, continued to use VM resources.

### Survey design and testing

A cross‐sectional survey was conducted to assess the use of VM among undergraduate medical and dental students at the UGMS who received histology instruction before, during, and after the COVID‐19 pandemic. The exact timeline is described in the previous paragraph. This survey specifically addressed the challenges for the use of VM among UG Medical/Dental School students during the COVID‐19 pandemic and how these students integrated VM as a supplemental resource into their learning process. A Qualtrics (Silver Lake, Seattle, WA, USA) survey was designed collaboratively with the student co‐author (S.N.O.) to ensure its relevance and clarity. Depending on whether the surveyed student used or did not use VM, the survey featured 20 or 6 questions, respectively. The full survey is available as a supplementary document from the publisher's website. Prior to implementation, the survey underwent a single‐cycle verification process with eight medical students from the Family Health Medical School, a private medical institution in Accra, Ghana. Feedback from this pilot study was used to refine the survey questions and structure. Additionally, the intrinsic quality control feature provided by the Qualtrics platform was employed to ensure data integrity and consistency.

### Participant recruitment and data collection

Participants were recruited from the general medical and dental student population at the University of Ghana (levels 200–600). Initial invitations to participate were disseminated via WhatsApp groups that are frequently used by the target demographic and by QR codes. These online invitations were further supplemented by personal outreach from both the histology instructors and the student co‐author, who extended invitations in person during classes, or via WhatsApp messages and direct emails. This multifaceted recruitment strategy was designed to maximize response rates and to ensure a representative sample. No survey incentives were offered.

### Data screening and analysis

In total, 630 responses were collected during the survey period from May 31, 2023 to July 16, 2023. These responses were subjected to a rigorous screening process to ensure eligibility. The exclusion criteria were incomplete submissions (*N* = 49), responses from individuals not enrolled at UG as a medical or dental student (*N* = 12), and duplicate entries (*N* = 16). After application of these exclusion criteria, 553 valid responses were anonymized and retained for further analysis.

Descriptive statistical methods like frequencies and percentages were employed to assess students' familiarity with and use of VM. The Pearson's chi‐square test was used to test for associations. In cases of sparse data, the Fischer's exact test was employed. The statistical analysis was conducted using STATA version 17.0 (StataCorp LLC, College Station, TX, USA).

This study was conducted in compliance with ethical standards as outlined by the Declaration of Helsinki and all participants provided informed consent prior to their participation. The project received the ethical clearance number UGMS‐CHDRC/154/2023 from the Ethics and Protocol Review Board of the Dissertation Review Committee, Department of Community Health, University of Ghana Medical School.

## RESULTS

### Responses by UG students to a survey about virtual microscopy

A total of 1156 UG students were invited to participate in the survey. Survey responses that came from non‐medical/non‐dental students were eliminated,^12^ as well as duplicate^16^ and incomplete survey submissions.^49^ A total of 553 valid and completed survey responses were collected from class levels 200–600 (Table 1). 83.3% were medical and 16.7% were dental students (Table 1). Female students had a slightly higher response rates than male students (Table 1). Similarly, medical students responded at a higher rate when compared to dental students (Table 1). Participation of students at different class levels varied from 31.9% to 72.6% with classes being enrolled in histology at the time of the survey (levels 200 and 300) answering at higher rates than classes that had completed their histology education (Table 1). Overall, the combined survey participation rate for all surveyed class levels was 47.8% (Table 1).

**TABLE 1:** Characteristics of students who responded to the survey.

Characteristics	Total number of students invited	Percent of all students that were invited to the survey	Number of completed survey responses	Percent of all completed survey responses	Response rate (%)
All students invited	1156		553		47.8
Gender:					
Female	593	51.3	300	54.3	50.6
Male	563	48.7	250	45.2	44.4
Prefer not to answer			3	0.5	
Field of study:					
Medical	963	83.3	473	85.5	49.1
Dental	193	16.7	80	14.5	41.5
Class level:					
200	190		138	24.9	72.6
300	236		122	22.1	51.7
400	259		106	19.1	40.9
500	238		76	13.7	31.9
600	233		111	20.1	47.6

### Knowledge about and use of online virtual microscopy resources

**FIGURE 1:**
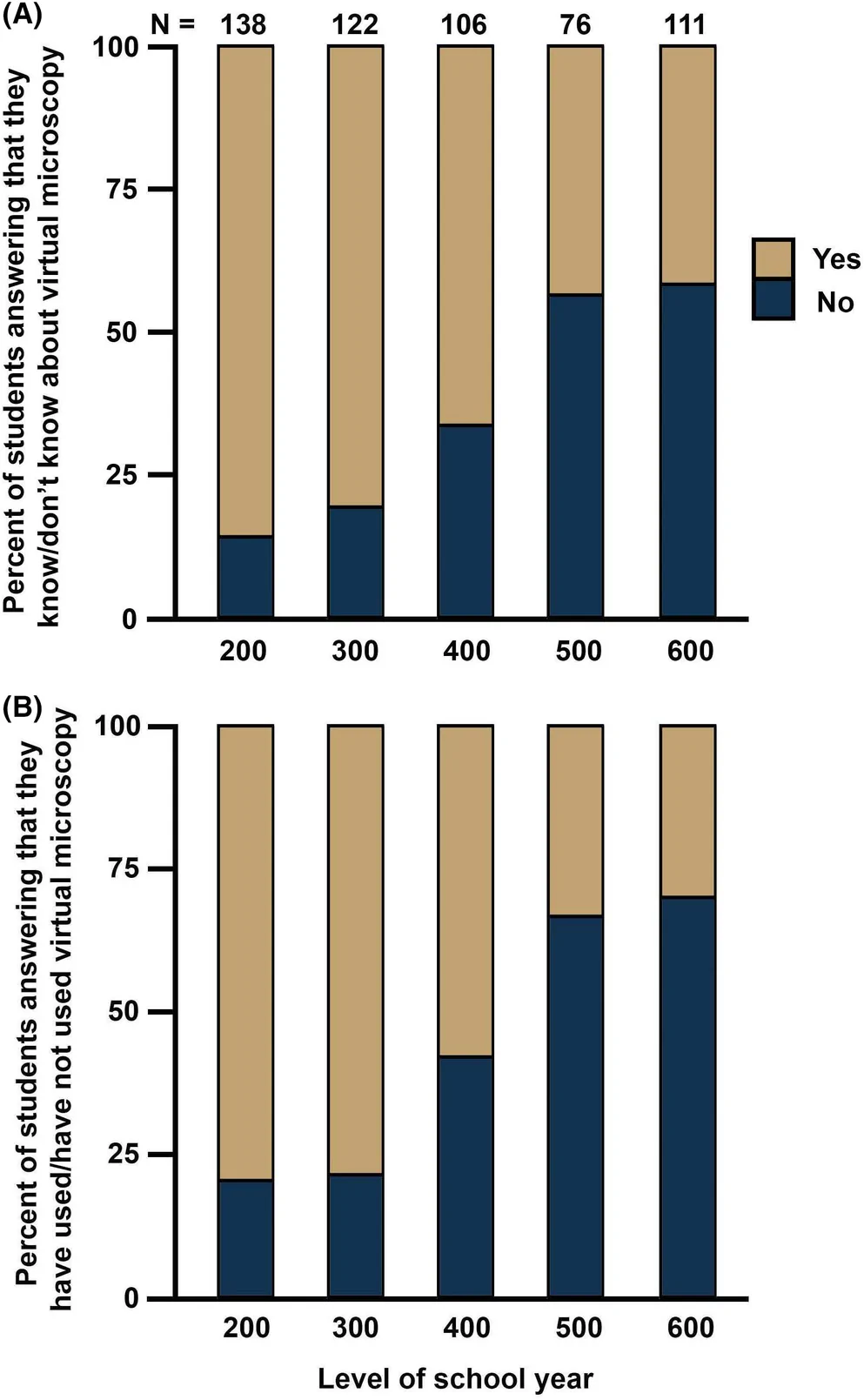
Changes of virtual microscopy awareness (A) and use (B) by medical and dental students at UGMS over a 5‐year period covering the COVID‐19 pandemic. Whereas the 600‐level class learned histology prior to the onset of the COVID‐19 pandemic in 2020, starting with class 500, all other classes were either directly affected by the pandemic or received histology instruction after the pandemic restrictions were lifted.

When survey participants were asked about their knowledge about VM, about two‐thirds of all participants were familiar with this technology, and slightly fewer students answered that they previously had used VM (Table 2). However, when analyzed by class level, a significant pattern of change for knowledge about and use of VM was observed (Figure 1). The 600‐level class took histology during pre‐pandemic times, and most 600‐level students were unfamiliar with VM with even more students who had not used the technology (Figure 1). The first class that received histology instruction during the onset of the COVID‐19 pandemic was the 500‐level class. Subsequent classes (200, 300, and 400) took histology classes during or past the pandemic and stated an increased awareness (chi‐squared test: *df* = 4; *χ* ^2^ = 81.6; *p*‐value < 0.0001) and use of VM (chi‐squared test: *df* = 4; *χ* ^2^ = 99.2; *p*‐value < 0.0001) (Figure 1).

**TABLE 2:** Knowledge about and use of online VM resources.

Knowledge indicators	Number of answers	Percent (%) of all answers
Do you know about virtual microscopy (*N* = 533)		
Yes	365	66.0
No	188	34.0
Have you used virtual microscopy (*N* = 533)		
Yes	322	58.2
No	231	41.8
Which website(s) do you regularly use to access virtual microscopy slides (select all that apply, 322 students)		
Histology Guide Website (Brelje & Sorensen, USA)	273	60.0
Michigan Histology Website (University of Michigan, USA)	145	31.9
Histology Lab Manual (Columbia University, USA)	26	5.7
Indiana University Virtual Microscopy (Indianapolis, USA)	11	2.4
Other histology websites, YouTube, Google searches	14	4.3
What devices do you use to access virtual microscopy (select all that apply, 322 students)		
Mobile phones	283	50.7
Personal computers	202	36.2
Computer tablets	67	12.0
Library computer	6	1.1
Locations where you access virtual microscopy slides (select all that apply, 322 students)		
During laboratory sessions	254	40.1
At home	226	35.7
During lectures	86	13.6
In the library	67	10.6

The 322 students, who answered that they had used VM, were asked several additional questions. As the University of Ghana does not offer any VM resources of its own, students were asked which online VM resources they frequently used. They were given four choices of online histology websites featuring VM slides and the option to fill in additional websites. Although not part of the official histology curriculum, these four websites were known to be popular among UG histology learners and were also recommended by the histology teaching staff. Most popular were the Histology Guide and the Michigan Histology websites^51^, ^52^ (Table 2). Additional histology and pathology websites that were mentioned by individual students included UCSD Medpics, Histology@Yale, Histology at Southern Illinois University, the University of Oklahoma Histology Atlas, and the Internet Pathology Laboratory from the University of Utah, as well as YouTube and Google searches. However, these websites, except for the Histology@Yale resource,^50^ only offer static histology images, not scalable VM histology slides. It is noteworthy that these websites are all offered by North American individuals or universities and are accessible without charge or registration.^52^

Survey participants, who had used VM, were also asked about the electronic devices and locations of their VM studies (Table 2). Smartphones and personal computers were used most frequently for studying VM slides (Table 2). Few students used computer tablets or library computers. VM resources were most often accessed during regular histology laboratory sessions or when studying at home (Table 2).

### Challenges for UG students' virtual microscopy use

**FIGURE 2:**
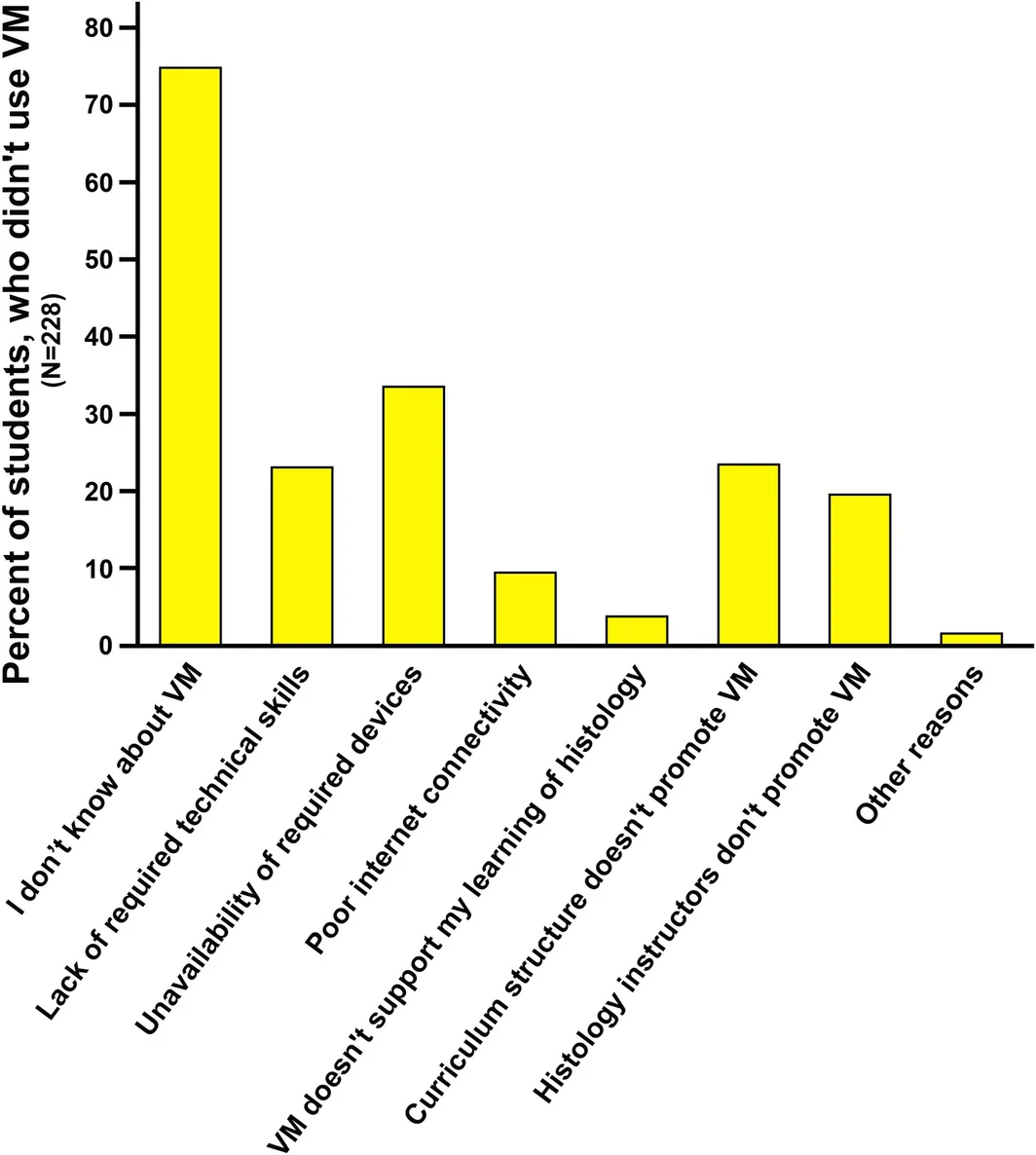
University of Ghana medical and dental students were asked for the main reasons why they did not use VM resources for their histology learning. Students were able to select multiple answers.

VM users were asked about challenges, which they encountered when using VM (Table 3). Most students cited internet access and its financial cost as the main obstacles when using VM. Relatively few surveyed VM users mentioned the lack of a personal device or unfamiliarity with the technology as an impediment for VM use. Other reasons that were mentioned included more general issues about VM technology, such as VM slides being unlabeled, that no teacher guidance was available when accessing VM websites, and unspecified technical problems. Non‐VM users were also asked for reasons that prevented them from using VM resources (Figure 2). Their main reasons for not using VM technology were unfamiliarity with VM and that these resources were not part of the curriculum and not sufficiently promoted by the instructional personnel. A second set of obstacles for their lack of VM use was the shortage of computing devices, technical knowledge, and poor internet connectivity (Figure 2).

**TABLE 3:** What are the challenges for you when using virtual microscopy for histology learning? (Select all that apply; 322 students).

Characteristics	Number	Percent (%) of VM users
No personal device to access the internet	6	1.8
Internet costs	157	46.9
Internet access (technical limitations)	124	37.0
Unfamiliar or uncomfortable to use this technology	48	14.3

### UG students' perceptions about virtual microscopy

A majority of students, who used VM, voiced the opinion that VM technology has many positive attributes for learning histology (Table 4). They felt that it saves time and was easy to use. In general, they attributed VM technology with a better understanding of the histology learning material and the development of their own scientific and analytical abilities. In the students' opinion, VM technology also fosters discussions between peers and teachers and solidifies histology knowledge. However, when asked whether VM could completely replace traditional microscopy for histology instruction, more than 70% of VM student users were either unsure or disagreed. When ask about the best technology for histology laboratory sessions, more than 90% of the students, who had VM experience, advocated the hybrid use of both traditional LM and VM (Table 5).

**TABLE 4:** Student opinions about using VM for histology learning (322 students).

Survey questions	Strongly agree percent (*N*)	Agree percent (*N*)	Not sure percent (*N*)	Disagree percent (*N*)	Strongly disagree percent (*N*)
Use of VM saves time	39.2 (126)	51.5 (166)	8.4 (27)	0.9 (3)	0.0 (0)
I clearly understand to use VM	27.9 (90)	58.7 (189)	11.2 (36)	1.9 (6)	0.3 (1)
It is easy to navigate virtual slides	27.6 (89)	60.9 (196)	7.5 (24)	3.7 (12)	0.3 (1)
It was easy to identify tissue details in VSs	26.1 (84)	55.3 (178)	12.1 (39)	5.9 (19)	0.6 (2)
I found it helpful to use VM outside laboratory sessions	39.4 (127)	52.5 (169)	5.6 (18)	1.9 (6)	0.6 (2)
VM helps to solidify my understanding of histology	39.1 (126)	55.3 (178)	5.3 (17)	0.3 (1)	0.0 (0)
VM makes it easier to discuss microscopic findings	36.9 (119)	53.4 (172)	8.4 (27)	1.2 (4)	0.0 (0)
Exposure to VM has helped to develop my scientific and analytical abilities	20.5 (66)	54.1 (174)	22.9 (74)	2.2 (7)	0.3 (1)
VM can completely replace traditional microscopy for learning histology	10.6 (34)	19.3 (62)	24.2 (78)	37.3 (120)	8.7 (28)

**TABLE 5:** In your opinion, what is the best technology for practical sessions to learn histology?

Characteristics	Number *N* = 322	Percent (%)
Traditional microscopy alone	3	0.9
Virtual microscopy alone	25	7.8
Combination of traditional and virtual microscopy	294	91.3
Static images, e.g., in textbooks or from other sources	0	0

## DISCUSSION

The introduction of e‐learning approaches and resources to professional school education is a global phenomenon.^1^, ^4^, ^60^ This includes the use of VM for histology and pathology laboratory instruction at medical, dental, veterinary, and other programs, replacing traditional LM as the main laboratory teaching technology.^3^, ^7^, ^19^ However, how far this conversion has progressed in different global regions differs widely, often correlating with the economic strength of the countries where individual schools are located.^2^, ^3^, ^4^, ^19^ Even within a single continent, such as Africa, the use of e‐learning technologies is very uneven, with universities in the Maghreb, Egypt, and South Africa utilizing VM more frequently than those in other African countries.^46^, ^47^, ^74^, ^75^ However, there are some reports describing the use of VM for the teaching of histology and pathology at several sub‐Saharan institutions, specifically in Zambia, Mozambique, Namibia, and Nigeria.^76^, ^77^, ^78^, ^79^ As these publications involve only a limited number of African universities, this does not reflect a widespread use of this educational technology at the present time.

At the beginning of the COVID‐19 pandemic, universities worldwide were challenged to quickly abandon in‐person instruction, often by utilizing e‐learning technologies.^55^, ^56^, ^58^, ^62^, ^80^, ^81^, ^82^ Students and educators in affluent countries relied on personal computing devices and existing modes of long‐distance learning, whereas learners and educators in developing countries often faced significant obstacles when adopting a non‐contact mode of instruction and learning. This included the use of VM for the laboratory components of histology and pathology instruction.^63^, ^66^, ^83^, ^84^

One major roadblock for the introduction of e‐learning strategies at universities located in developing countries is the access to personal computers or tablets.^36^, ^37^, ^62^, ^80^, ^85^ According to a 2019 government survey, less than 7% of the Ghanaian population owned a computer (laptop or desktop) or computer tablet.^86^ Student responses in Table 2 suggest that computer ownership among UG students might be higher than for the general population in Ghana. Nevertheless, most histology learners at UGMS used their smartphone for accessing VM resources (Table 2). This indicates a resourcefulness and resilience by learners when confronted with technical obstacles during the adoption of new e‐learning strategies. Smartphones are widely available in Ghana^86^ and in many other developing countries where they are often used for the learning of histology/pathology and other biomedical topics.^57^, ^87^, ^88^, ^89^, ^90^, ^91^, ^92^ In contrast, in affluent countries like the United States, where personal computer ownership is high, very few students use their smartphone as the primary device for higher education learning.^93^ The major uses of smartphones among medical students in affluent countries are more specific and include mobile medical education apps and online access to clinical information resources.^94^, ^95^, ^96^

A second obstacle for the use of VM for histology education is fast and reliable access to the internet. Nikas et al. reported that the quality of the students' internet connection correlated with final examination scores for both histology and pathology.^68^ Poor internet connectivity is a problem in Ghana and most other countries in Africa.^80^, ^85^ Broadband mobile internet access in Ghana is improving, specifically in urban areas, but remains unreliable. It is slow and is generally accessed using smartphones.^97^ Lack of personal computing devices and of internet access were both named as major obstacles to VM use by UGMS non‐VM users (Figure 2). In contrast, UG students, who used VM for histology learning, mostly named the availability of internet access and its cost as impediments for their VM usage. Access to a personal computer/tablet was mentioned by few students as a hindrance for using VM (Table 3). Thus, there is a heterogeneity among UGMS histology learners whether both or only one of these aspects limited their VM use.

With limited access to personal computer devices and unreliable internet connectivity, it was surprising that UG medical and dental students did not make more use of university computing facilities (Table 2). This may be due to two major reasons. Although the University of Ghana has a computer facility on its main campus, the Medical School is located on the Korle‐Bu campus, 20 miles away. At the Korle Bu campus, medical and dental students only have access to a small computer lab (16 computers), which is also shared with School of Biomedical and Allied Health Sciences students. Moreover, during the COVID‐19 pandemic, this computer facility was closed and not accessible to UGMS learners. Second, the current medical/dental curriculum at the University of Ghana does not schedule time for self‐studying, thereby restricting the time for online learning to official instruction sessions like lectures or histology laboratories or when students are at home (see Table 2). Providing better computing facilities to more students would be helpful for introducing additional e‐learning elements into the UGMS curricular structure. However, this requires a major space and financial investment by the university. As a first step, in 2024, an additional 40 computer stations were added to the UGMS computer facility.

With some exceptions, students are usually open to the use of novel teaching and learning technologies.^62^, ^82^, ^92^, ^98^, ^99^ Among UG students, who experienced VM, the perception of this learning technology is overall positive (Table 4). However, even these students overwhelmingly (>90%) advocate for the hybrid use of traditional LM and VM in histology and pathology teaching laboratories (Table 5). Many previous studies reported that histology and pathology learners usually prefer the use of VM over LM.^26^, ^99^, ^100^, ^101^, ^102^ However, UGMS and UGDS students are not alone in supporting the use of both technologies for histology laboratory education.^103^, ^104^, ^105^

One additional important factor that was identified in this study was the need for the active promotion of new learning technologies by the teaching and administrative staff (Table 2). In pre‐pandemic times, the survey results indicate a low level of VM awareness among UG histology learners, resulting in an even lower use of this technology (Figure 1). Although the pandemic‐induced changes to histology laboratory teaching at UGMS were relatively short term, they resulted in significant permanent changes in students' familiarity and usage of VM, even after the return to the pre‐pandemic mode of histology teaching. Some of the UGMS teaching faculty members are actively promoting the use of VM resources to UG histology learners. However, it is often the administrative staff that exhibits hesitations when adopting new teaching and learning technologies. This can be attributed to economic, practical and administrative constraints, roadblocks that are also present in many other developing countries.^40^, ^44^, ^47^, ^48^, ^62^, ^106^ A report from South Africa indicates that educators are often reluctant or unable to effectively integrate these new technologies into their teaching.^107^ Awareness by and support from the teaching community is only one of several factors that will make a switch toward the use of e‐learning strategies successful.^108^, ^109^ In addition, the curricular integration of VM into medical and dental school teaching will help facilitate a smoother transition to a more effective use of e‐learning technologies. This requires active participation from the university and school's administration to incorporate e‐learning into the curricular structure and to provide student access to computing devices, the internet, and electronic learning resources.

Like other African countries that were part of the British colonial empire, English is the official language in Ghana for governmental affairs, business, and education. As open educational resources on the internet are usually in English, it makes the adoption of foreign learning tools easier for Ghanaian students. Students in countries where English is not the primary language of instructions face an additional language barrier when using these tools.^110^, ^111^ Most online VM resources are offered by individuals or institutions in affluent countries, primarily the United States, Europe, and Australia^1^, ^52^, and may not always be organized in a way to effectively serve local educational needs. In the long term, educational institutions in developing countries may want to develop their own e‐learning resources to minimize the dependence on foreign educational tools. Even if that is currently not a feasible path, the use of free image and resource sharing databases may provide a first step toward eventually reaching this goal.^29^, ^112^

Reports from other African and non‐African developing countries mirror the findings reported in this analysis, specifically a general acceptance of new educational technologies by students, a strong reliance on personal computing devices, especially smartphones, and a lack of curricular integration and promotion by the educational staff.^2^, ^40^, ^41^, ^62^, ^88^, ^113^ For countries with limited educational resources, O'Doherty et al. summarized the key barriers for the development and implementation of online learning in medical education with time constraints, poor technical skills, inadequate infrastructure, an absence of institutional strategies and support, and negative attitudes of learners, teachers, and administrators.^114^

The urgency for the introduction of e‐learning technologies like VM is growing, especially in developing regions of the world. There is a desperate need for more trained medical/dental professionals in Ghana and many other countries.^115^, ^116^ As a response to that need, new universities with biomedical programs are founded in Africa and in other regions of the world.^117^, ^118^ The resulting surge in the number of students requiring an education in the anatomical sciences is further straining the existing limited resources. VM and other e‐learning approaches may help to accommodate this increasing number of learners.^62^, ^117^, ^119^, ^120^

This study identified several significant barriers for the adoption of VM, specifically a lack of personal computing devices, limited internet access and its high cost, which impede students' ability to utilize digital slides outside of laboratory sessions, as well as a lack of institutional promotion of its use in education. These challenges are not unique to Ghana or Africa. For example, in Brazil and India, social inequalities and technological disparities similarly affect the widespread adoption of VM for medical education.^40^, ^41^, ^88^ Addressing these infrastructural and economic constraints is essential for the successful adoption of VM into histology and pathology education. Among others, potential solutions may include the integration into the curricular structure as well as institutional support and investments in on‐campus computer facilities with internet access. Partnerships with technology providers and/or governmental agencies to subsidize costs for students' personal computing devices and internet access would also be beneficial. International collaboration may also provide a helpful strategy in moving anatomical science education forward at African and other schools.^121^, ^122^, ^123^

### Study limitations

The reported results were obtained from students at a single African university, the University of Ghana in Accra, and therefore may not fully apply to other universities in sub‐Saharan Africa or other developing countries. The survey that provided the data for analysis was only offered online as a Qualtrics questionnaire. This may have excluded some students who lack internet access or an appropriate device to participate in this type of survey. Despite the definition given at the beginning of the survey, a few students may have considered static histology images that are delivered via the internet as VM.

## CONCLUSIONS

This study highlights the potential of electronic learning resources like VM for enhancing histology education at UGMS and other universities in developing countries and for dealing with teaching emergencies like the COVID‐19 pandemic. However, the introduction of such educational strategies into resource‐challenged learning environments is complex and is facilitated or hindered by multiple factors, an affordable access to the internet and the availability of personal or school‐provided computers/tablets being only two infrastructural aspects. Overcoming traditional teaching paradigms and embracing newer ways of teaching histology and other basic sciences by learners, the teaching staff, and the university administration are equally important factors. A strategic, blended approach that integrates the strengths of both traditional LM and VM and that is supported by institutional commitments and investments is recommended to optimize histology education, not only at UGMS but also in other similar educational settings.

## AUTHOR CONTRIBUTIONS

**Nii Koney‐Kwaku Koney:** Conceptualization; methodology; supervision; project administration; writing – original draft; writing – review and editing. **Smart Nana Omenako:** Conceptualization; methodology; project administration; writing – review and editing. **Michael Hortsch:** Investigation; project administration; visualization; supervision; writing – original draft; writing – review and editing. **David Nana Adjei:** Formal analysis; writing – review and editing; writing – original draft. **Benjamin Arko‐Boham:** Conceptualization; methodology; writing – review and editing.

## CONFLICT OF INTEREST STATEMENT

The authors report no conflicts of interest.

## Supporting information


Data S1.

## Data Availability

The data that support the findings of this study are available on request from the corresponding author. The data are not publicly available due to privacy or ethical restrictions.
