# Supplementary material for: Introducing virtual microscopy at the University of Ghana during the COVID‐19 pandemic—Identifying and overcoming obstacles in a low‐resource learning environment
Source: Anat Sci Educ. 2026 Jun 8;19(9):1438–51. doi: 10.1002/ase.70276 (PMC13545326; doi:10.1002/ase.70276)
Supplement: Supplementary file 1 [file ASE-19-1438-s001.docx]

I am SMART NANA OMENAKO a final year student at the UNIVERSITY OF GHANA undertaking research on the topic of *ASSESSING THE USE AND IMPACT OF VIRTUAL MICROSCOPY IN AN UNDERGRADUATE HISTOLOGY COURSE AT THE UNIVERSITY OF GHANA MEDICAL AND DENTAL SCHOOLS.*

The aim of this study is to evaluate how students at the University of Ghana Medical/Dental School use and value virtual histology images for their education. For the purpose of this survey, **virtual microscopy (VM)** is defined as a technology that digitalizes microscopic histology or pathology slides into high resolution computer files that can be viewed at different magnifications, as well as analyzed and shared using smartphones, computers and other electronic devices via computer networks such as the internet. It provides an alternative to **traditional optical microscopes and glass slides (TM)**.

## **SPECIFIC OBJECTIVES**

1. To determine the preferred way of studying histology among University of Ghana medical and dental students
2. To identify the barriers for the use of virtual microscopy among University of Ghana Medical/Dental School students.
3. To determine the preferred choice of devices for accessing virtual microscopy
4. To determine the challenges associated with using virtual microscopy.

The research is solely for academic purposes. Aggregated results and anonymous comments may be published in an academic journal.

Privacy and confidential of participants will be strongly upheld. In no way will the participation in this research study affect participants either negatively or positively.

ETHICAL CLEARANCE NUMBER: UGMS-CHDRC/154/2023.

This questionnaire is organized in six sections and should take no longer than 10 minutes to complete. For each question, please tick the response(s) most applicable to you. Please submit only one set of answers per person/student.

**CONSENT FORM**

A STUDY ASSESSING THE USE AND IMPACT OF VIRTUAL MICROSCOPY IN AN UNDERGRADUATE HISTOLOGY COURSE AT THE UNIVERSITY OF GHANA MEDICAL AND DENTAL SCHOOLS. My name is SMART NANA OMENAKO, a final year medical student of the University of Ghana Medical School. I am conducting a study on the topic above in partial fulfilment of the requirements for the award of a Bachelor of Medicine, Bachelor of Surgery degree (MBChB). I would be very grateful if you would participate in this study by filling out this questionnaire. Your participation is voluntary, and you can decide to withdraw from this study at any point in time. You are not required to disclose your identity and confidentiality is assured.

I (signature below) have read and understood the information provided above and willingly agree to participate, fully assured that any information disclosed in this questionnaire would remain confidential.

Signature………………………

Date……………………………

1. **PERSONAL DATA**
   1.1 Gender

[	] Male

[	] Female

[	] Prefer not to answer

1.2 Are you a medical or dental student?

[	] Medial

[	] Dental

[	] Other (not medical, not dental)

1.3 What is your current level/year of medical or dental school?

[	] 100

[	] 200

[	] 300

[	] 400

[	] 500

[	] 600

**PART 2: ACCESSIBILITY OF VIRTUAL MICROSCOPY**

2.1 Do you know about virtual microscopy and how it is used for learning histology?

[	] Yes

[	] No.

2.2 Have you used virtual microscopy to study histology?

[	] Yes

[	] No

If your answer is YES, kindly continue with the next question. If your answer is NO, please skip to PART 6.

2.3 Which website(s) do you regularly use to access virtual microscopy slides? (Check all that apply).

[	] University of Michigan website.

[	] Histology Guide

[	] Indiana University School of Medicine Virtual Microscopy

[	] Histology Lab Manual (Columbia University)

Any other site ………………………………….

2.4 What devices do you use to access virtual microscopy. (Check all that apply)

[	] Library computers

[	] Personal computers

[	] Mobile phones

[	] Computer tablets

Other: …………………….

2.5 If you regularly use a mobile phone for learning histology, what brand do you use?

[	] iPhone

[	] Samsung

[	] Huawei

[	] Infinix

Other ……………………

2.6 Where do you usually access virtual microscopy slides? (Check all that apply)

[	] During laboratory sessions

[	] At home

[	] During lectures

[	] In the library

[	] Other ………………

**PART 3: LEARNING EFFICIENCY**

3.1 The use of virtual microscopy is saving time. (Select only one answer)

[	] Strongly agree

[	] Agree

[	] Not sure

[	] Disagree

[	] Strongly disagree

3.2 I clearly understand how to use virtual microscopy. (Select only one answer)

[	] Strongly agree

[	] Agree

[	] Not sure

[	] Disagree

[	] Strongly disagree

3.3 It is easy to navigate through the virtual slides. (Select only one answer)

[	] Strongly agree

[	] Agree

[	] Not sure

[	] Disagree

[	] Strongly disagree

3.4 It was easy for me to identify tissue details in virtual slides. (Select only one answer)

[	] Strongly Agree

[	] Agree

[	] Not sure

[	] Disagree

[	] Strongly disagree

3.5 I found it helpful to use virtual microscopy outside of scheduled laboratory hours. (Select only one answer)

[	] Strongly agree

[	] Agree

[	] Not sure

[	] Disagree

[	] Strongly disagree

3.6 What are the challenges for you to using virtual microscopy for histology learning? (Select all that apply)

[ ] Internet costs

[ ] No personal device to access the internet

[ ] Internet access (technical limitations)

[ ] Unfamiliar or uncomfortable to use this technology

Other ………………………….

**PART 4: KNOWLEDGE ACQUISITION**

4.1 Studying histology with virtual microscopy helps solidify my understanding of histology? (Select only one answer)

[	] Strongly agree

[	] Agree

[	] Not sure

[	] Disagree

[	] Strongly disagree

4.2 Virtual microscopy makes it easier to discuss microscopic findings with other students and teachers. (Select only one answer)

[	] Strongly agree

[	] Agree

[	] Not sure

[	] Disagree

[	] Strongly disagree

4.3 Exposure to virtual microscopy helps me to develop my ability to think scientifically and analytically. (Select only one answer)

[	] Strongly agree

[	] Agree

[	] Not sure

[	] Disagree

[	] Strongly disagree

**PART 5: COMPARING VIRTUAL WITH TRADITIONAL MICROSCOPY**

5.1 In your opinion, what is the best technology for practical sessions to learn histology? (Select only one answer)

[	] Traditional microscopy alone

[	] Virtual microscopy alone

[	] Combined traditional and virtual microscopy

[	] Static images, e.g., in textbooks or from other sources

[	] None of the above

5.2 Do you think that virtual microscopy can completely replace traditional microscopy for the learning of histology? (Select only one answer)

[	] Strongly agree

[	] Agree

[	] Not sure

[	] Disagree

[	] Strongly disagree

**PART 6: REASONS FOR NOT USING VIRTUAL MICROSCOPY**

6.1 What reason do you have for not using virtual microscopy for histology learning? (Select all that apply)

[	] Don’t know about virtual microscopy

[	] Lack of required technical skill

[	] Unavailability of required devices

[	] Poor internet connectivity

[	} Virtual microscopy does not support my learning of histology

[	} The curriculum structure does not promote the use of virtual microscopy.

[	] The histology instructors do not promote the use of virtual microscopy.

Any other reason ……………………………………

We thank you for your time spent taking this survey.
